# Scrutinizing the Feasibility of Nonionic Surfactants to Form Isotropic Bicelles of Curcumin: a Potential Antiviral Candidate Against COVID-19

**DOI:** 10.1208/s12249-021-02197-2

**Published:** 2021-12-29

**Authors:** Dina B. Mahmoud, Mohamed Mofreh Bakr, Ahmed A. Al-karmalawy, Yassmin Moatasim, Ahmed El Taweel, Ahmed Mostafa

**Affiliations:** 1 Department of Pharmaceutics, Egyptian drug Authority (formerly known as National Organization for Drug Control and Research), Giza, Egypt; 2 Pharmaceutical Technology, Institute of Pharmacy, Leipzig University, 04317 Leipzig, Germany; 3 Department of Pharmaceutical Medicinal Chemistry, Faculty of Pharmacy, Horus University-Egypt, New Damietta, 34518 Egypt; 4 Center of Scientific Excellence for Influenza Viruses, National Research Centre, Giza, 12622 Egypt

**Keywords:** Bicelles, Mixed micelles, Molecular docking, Non-ionic surfactants, SARS-CoV-2

## Abstract

Investigating bicelles as an oral drug delivery system and exploiting their structural benefits can pave the way to formulate hydrophobic drugs and potentiate their activity. Herein, the ability of non-ionic surfactants (labrasol^®^, tween 80, cremophore EL and pluronic F127) to form curcumin loaded bicelles with phosphatidylcholine, utilizing a simple method, was investigated. Molecular docking was used to understand the mechanism of bicelles formation. The % transmittance and TEM exhibited bicelles formation with labrasol^®^ and tween 80, while cremophor EL and pluronic F127 tended to form mixed micelles. The surfactant-based nanostructures significantly improved curcumin dissolution (99.2 ± 2.6% within 10 min in case of tween 80-based bicelles) compared to liposomes and curcumin suspension in non-sink conditions. The prepared formulations improved curcumin *ex vivo* permeation over liposomes and drug suspension. Further, the therapeutic antiviral activity of the formulated curcumin against SARS-CoV-2 was potentiated over drug suspension. Although both Labrasol^®^ and tween 80 bicelles could form bicelles and enhance the oral delivery of curcumin when compared to liposomes and drug suspension, the mixed micelles formulations depicted superiority than bicelles formulations. Our findings provide promising formulations that can be utilized for further preclinical and clinical studies of curcumin as an antiviral therapy for COVID-19 patients.

**Figure:**
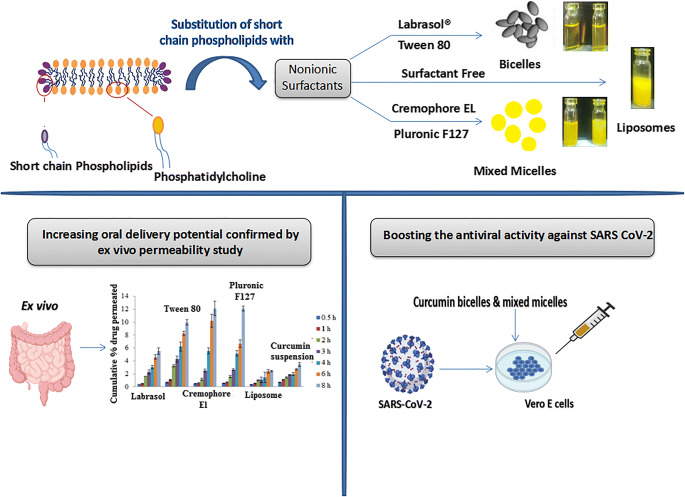
Graphical Abstract

Graphical Abstract

## INTRODUCTION

Bicelles are lipid bi-layered nanodiscs, that usually formed as mixtures of long chain phospholipids along with short chain phospholipids (1). The morphology of the bicelles comprises a planar bilayer disk (long chain phospholipids) with a rim of short chain phospholipids which shield the hydrophobic tails of the long chain phospholipids from water (2). Hydrophilic surfactants such as dodecyltrimethylammonium chloride (3) and tween 80 (4) could also be used as substitutes for short chain phospholipids for the formulation of bicelles. Particularly, nonionic surfactants for instance tween 80 possess much lower critical micellar concentrations either than ionic surfactants or short chain phospholipids, which is favorable for efficient bicelle formation. Moreover, mixtures of long chain phospholipid with a nonionic surfactant depict an improved bicelles stability because of the denser packing of hydrophobic tails resulting from moderate repulsions between the hydrophilic head groups (5). Various applications of bicelles have been discussed in literature such as their use in protein structure studies (6), membrane protein crystallization (7), cell membrane models (8) and nanomaterial synthesis (9). Besides, their applications as coating materials (10) and carriers for gene therapy (11,12) or drug delivery to the skin (13). The structure of bicelles imparts them the ability to encapsulate different compounds and deliver them to the skin layers. Moreover, they possess smaller size when compared to liposomes with higher penetration ability through the tight intercellular spaces of the stratum corneum (14). Though, there are limited reports in literature discussing the use of bicelles as drug delivery systems, the reported techniques for preparation of bicelles involved complicated methods such as thin film formation with multiple ultrasonication stages (13). Researchers have also reported semi-spontaneous simpler method utilizing the use of organic solvents and homogenization (5,15). However, the potential use of bicelles in drug delivery as well as searching for alternative components and easier preparation methods still need to be extensively studied.

Severe Acute Respiratory Syndrome Coronavirus-2 (SARS-CoV-2), is a positive single stranded RNA virus. (Itt) has recently emerged by the end of 2019 and it has been regarded as a pandemic, that threatens the global public health, by the World Health Organization (WHO). Countless and urgent responses have been initiated throughout the world in order to outline novel or repurposed medications against SARS-CoV-2 infection. Curcumin exerts diverse therapeutic actions including but not limited to antioxidant, anti-inflammatory, anticancer and antiviral actions (16,17). Several studies reported the potential use of curcumin for the treatment of respiratory tract viral infections (18,19), including coronaviruses (20). Recently, *in silico* studies have reported the efficacy of curcumin against SARS-CoV-2 through interaction with the virus enzymes (21,22). Curcumin has been reported to potentially hinder virus entry and replication; it can alleviate the clinical manifestations associated with SARS-CoV-2 infections as it repairs the damaged pneumocytes, cardiomyocytes and renal cells. Further, it has the potential to modulate the cytokines storms and lung inflammation (23). Though the potential benefits of using curcumin as a therapy for SARS-CoV-2 infections; its medical use maybe limited. Unfortunately, the extremely low aqueous solubility of curcumin (0.456 μg/ml) (24) restricts its effective oral delivery. Herein, our aim in this work is to investigate the viability of using nonionic surfactants for bicelles formulation utilizing simple formulation method. Computational techniques in drug design became very important not only for the discovery of new drugs but also for proposing and understanding variable mechanisms of actions for many drugs and compounds as well. Especially molecular docking strategy represents the most crucial method related to the aforementioned techniques (25–27). Hence, we aimed to use molecular docking to provide a better understanding of the potential interaction between the formulations constituents during bicelles formation. Further, we aimed to assess the feasibility of using bicelles as an oral drug delivery system to improve the delivery of hydrophobic drugs such as curcumin and enhance its therapeutic activity against SARS-CoV-2. To the best of our knowledge, there are no currently available reports discussing the use of nonionic surfactant based bicelles as an oral drug delivery system.

## MATERIALS AND METHODS

### Materials

Curcumin was kindly obtained by Medizen Pharmaceutical Industries, Alexandria, Egypt. Egg l-α-phosphatidylcholine, Cremophore EL (CrEL)(HLB=12–14), Tween 80 (T80) (HLB=15) and Pluronic F127 (PF127) were supplied from Sigma-Aldrich, St. Louis, USA. Labrasol^®^ (caprylocaproyl polyoxyl-8 glycerides NF, HLB=14) was kindly obtained as a gift from Gattefosse, France.

### Cells

Vero E6 cells were cultured in Dulbecco’s modified Eagle’s medium (Lonza, Verviers, Belgium) supplemented with 10% fetal bovine serum (Gibco, NY, USA), and 1% antibiotic antimycotic mixture (Lonza). Incubation of the cells was done at 37^°^C in a humidified 5% CO_2_ atmosphere. An hCoV-19/Egypt/NRC-3/2020 SARS-CoV-2 virus (Accession Number on GSAID: EPI_ISL_430820) was propagated in Vero-E6 cells and harvested after cytopathic effects (CPE) appearance. Viral stock was titred using plaque infectivity assay and stored at −80^°^C.

### Preparation of Curcumin-Loaded Nanostructures

Mixtures of 200 mg egg l-α-phosphatidylcholine and 1000 mg of each nonionic surfactant, as displayed in Table 1, were melted in a shaking water-bath (80^°^C), 10 mg of curcumin was dissolved in the molten mixture and then 10 mL deionized water (preheated at 80^°^C) was added drop-wise to the molten mixture with stirring at 750 rpm for 30 min. The ratio of phosphatidylcholine, surfactant and drug in the formulation was kept at 1: 5: 0.05, respectively. Finally, the prepared formulations were kept at ambient temperature for further characterization.

**Table 1:** Experimental Preparations of the Different Curcumin Nanostructures

#	Type of surfactant *	PS(nm)	PDI	ZP(mV)	Transmittance (%)
F1	Labrasol^®^	187.5 ± 31	0.462±0.003	-6.8±0.5	91.6±1.1
F2	T80	416.4 ± 30.8	0.458±0.026	-18.1±1.8	48.5±2.8
F3	CrEL	729.5 ± 85.7	0.639±0.066	-18.8±0.2	12.9±0.6
F4	PF127	1167.3 ± 53.4	0.914±0.041	-15.4±0.4	1.8±0.2
F5	--------	736.3 ± 39.5	0.818±0.025	-22.0±0.2	1.8±0.5

*Amounts of phosphatidylcholine, surfactant and curcumin were kept constant in all formulations as follows: 200 mg, 1000 mg, and 10 mg in 10 mL water, respectively

### Docking Studies

The four nonionic surfactants were subjected individually to molecular docking studies using MOE 2019.012 suite (28) to study their interactions with phosphatidylcholine during bicelles formation.

#### Preparation of the Four Nonionic Surfactants and Phosphatidylcholine

**Fig. 1:**
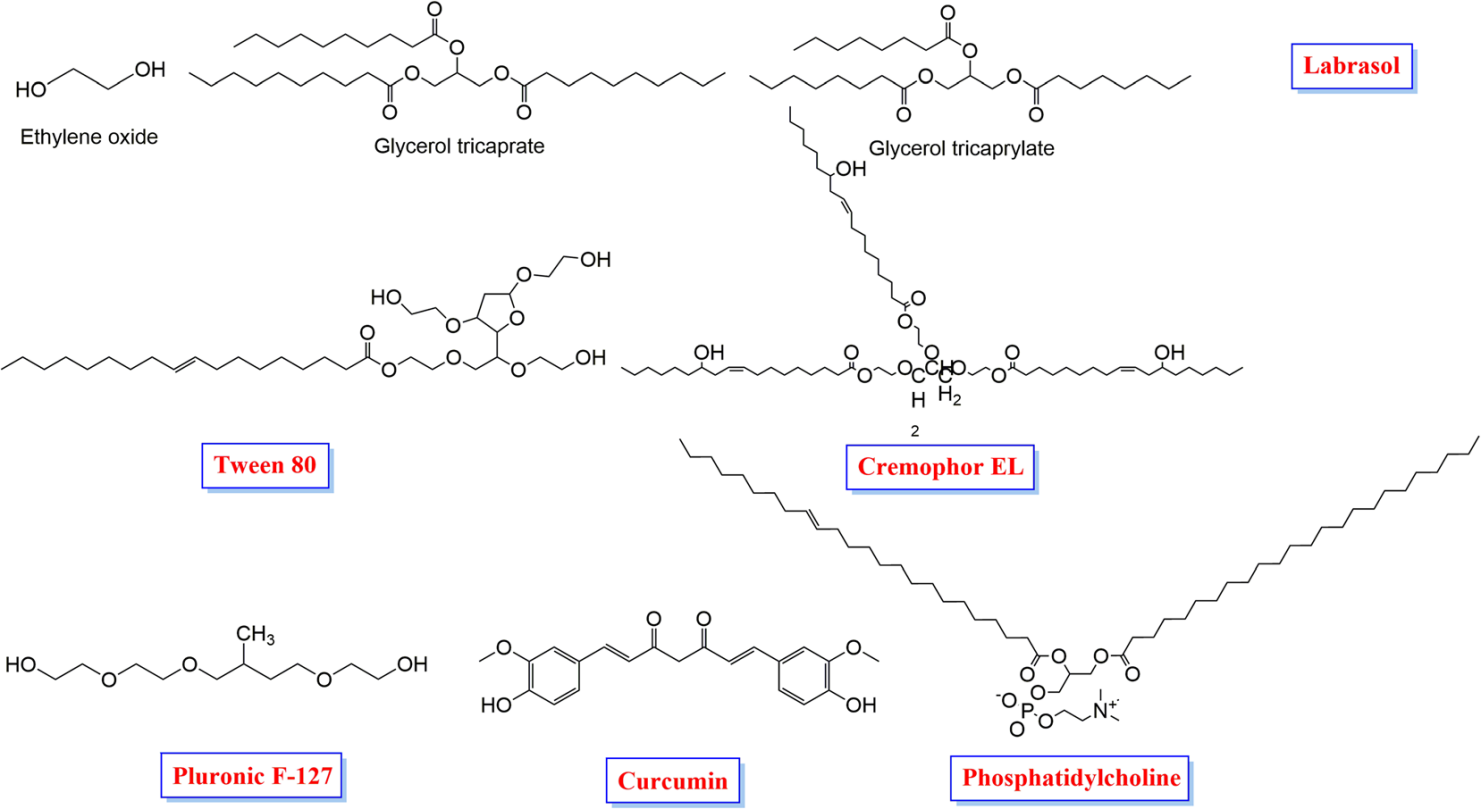
Chemical structures of Labrasol^®^ components, T80, CrEL, PF127, phosphatidylcholine and curcumin

The chemical structures of phosphatidylcholine and T80 were downloaded from the PubChem website (https://pubchem.ncbi.nlm.nih.gov/). Labrasol^®^, CrEL, and PF127 were sketched using the ChemDraw program (Fig. 1) (N.B. The components of Labrasol^®^ including poly(ethylene oxide), glycerol tricaprate, and glycerol tricaprylate were handled as three different components). Then, all of the previously mentioned chemical structures were imported one-by-one in the MOE program, subjected to partial charges formation and energy minimization processes as described earlier in detail (29,30). Each one of the previously prepared compounds was imported individually in a separate database and saved as an MDB file to be uploaded during the corresponding docking process for each.

#### Docking of Each Nonionic Surfactant Database to the Phosphatidylcholine

Each one of the previously prepared databases for the examined four nonionic surfactants (Labrasol^®^, T80, CrEL, and PF127) was docked separately to phosphatidylcholine database in a general docking process, respectively. The program specifications were adjusted according to the default steps discussed before (31,32) and the receptor site was selected to be (all atoms) to operate the molecular docking process for each. The best pose describing the interaction for each non-ionic surfactant with phosphatidylcholine was selected for deep evaluation.

### Chatacterization of the Prepared Nanostructures

#### Transmission Electron Microscopy (TEM)

To examine the morphological characteristics of the formed nanostructures, each formula was negatively stained by 2% w/v phosphotungistic acid. A one drop of diluted formulations was mounted on carbon coated copper grid, stained, dried at room temperature and examined by TEM (33).

#### Particle size (PS), Polydispersity Index (PDI), and Zeta Potential (ZP)

A Zetasizer (Nano ZS-90, Malvern instruments, UK) was utilized to determine PS, PDI and ZP of the prepared curcumin loaded nanostructures using dynamic light scattering technique. All samples were diluted with deionized water (100 times) and sonicated for 2 min. For ZP determination, a folded capillary zeta cell was used. All determinations were done in triplicates and average values ± SD were expressed.

#### Assessment of Transmittance Percentage

In order to determine the viability of the investigated non-ionic surfactants to form isotropic bicelles, the transparency of the formulations was judged by measuring the transmittance percentage by an ultraviolet-visible spectrophotometer (UV 1600, Shimadzu, Japan) at 650 nm using the deionized water as a blank (34).

#### *In Vitro* Dissolution Study

The dissolution of curcumin from the prepared nanostructures was performed using USP type II dissolution apparatus (Hanson Research, USA) which was rotated at 50 rpm. The bath temperature was kept at 37^°^C. In order to determine the ability of the formulations to enhance curcumin dissolution, non-sink condition was selected to perform the study; at which water was used as a discriminative dissolution medium. Briefly, a sample equivalent to 3 mg from each formulation was introduced into 300 mL of the dissolution medium and aliquots of 0.5 mL were taken at time intervals of (5, 10, 15, 30, 45 and 60 min), filtered through 0.2 μm PTFE filter and quantified for curcumin content by validated HPLC method utilizing (Agilent-HPLC1260, USA), mobile phase composed of acetonitrile and 2% v/v acetic acid (50:50) pumped at 0.6 mL/min, C18 Column (5 μm, 3mm X 30mm) kept at 35^°^C, 20 μL injection volume and the detection wave length was 425 nm.

#### *Ex Vivo* Permeability Test (Everted Gut Sac Model)

*Ex vivo* permeability test was performed using small intestine of rabbits that were allowed to fast overnight. The duodenal part was separated, flushed with normal saline to remove its content and portioned into segment sacs of 5 cm. Samples of 1mL of each formulation and the suspension of the crude drug, equivalent to 1 mg, was filled into each sac which their ends were tied carefully with sutures. Each sac was placed into a conical flask filled with 50 ml phosphate buffer pH 6.8 with 0.5% w/v SLS. All flasks were placed in a water bath shaker programmed at 100 rpm and 37 ^°^C (35). Aliquots of 1 mL were taken at time intervals of (0.5, 1, 2, 3, 4, 6 and 8h) and assayed by HPLC.

#### *In Vitro* Cytotoxicity and Antiviral Activity Against SARS-CoV-2

SARS-CoV-2 virus hCoV-19/Egypt/NRC-3/2020 isolate was titrated using Vero-E6 cells (ATCC, CRL-1586) under biological safety level 3 containment (36). Serially diluted virus was used to infect Vero-E6 cells post confluency into 96-well tissue culture plates. Cells were seeded in a 96-well plate and incubated under 5% CO_2_ condition in a humidified 37^°^C incubator. Cells monolayer was then washed twice with 1× PBS and infected with the serially diluted virus for 72 h at 37^°^C. Cell monolayers were then fixed with 3% paraformaldehyde and stained with crystal violet (0.1%). Virus titer was then calculated using Reed and Munch equation.

The cytotoxicity (CC_50_) was determined according to the protocol reported by Feoktistova et al. (37), serial dilutions of each formulation and control drug were used to treat Vero-E6 cells monolayers in 96-wells plate. At 72 h post treatment, cells were fixed and stained with crystal violet and then viability was measured as previously described in virus titration section. The CC_50_ of each investigated formulation which is the concentration required to cause the death of 50% of the seeded cells was determined and compared to the CC_50_ control untreated cells.

The IC_50_ was determined according to the protocol reported by Feoktistova et al. (37), with minor modification under biological safety level 3 (38). Serial dilutions of each formula and control drug were mixed with equal volume of 100 tissue culture infectious dose (TCID50/mL) and incubated for 1 h at 37^°^C. A volume of 100 μL of the virus–drug mix was overlaid in triplicate to Vero-E6 cell in a 96-well tissue culture plates and incubated at 37^°^C in 5% CO_2_ incubator for 72 h. cells were fixed with 4% paraformaldehyde and stained with 0.1% crystal violet. After dissolving the stain with methanol, the optical density of the color was measured at 570 nm. The IC_50_ of each investigated formulation which is the concentration required to reduce the virus-induced cytopathic effect (CPE) by 50% was assessed relative to virus control.

## RESULTS AND DISCUSSION

### Preparation of Curcumin-Loaded Nanostructures

**Fig. 2:**
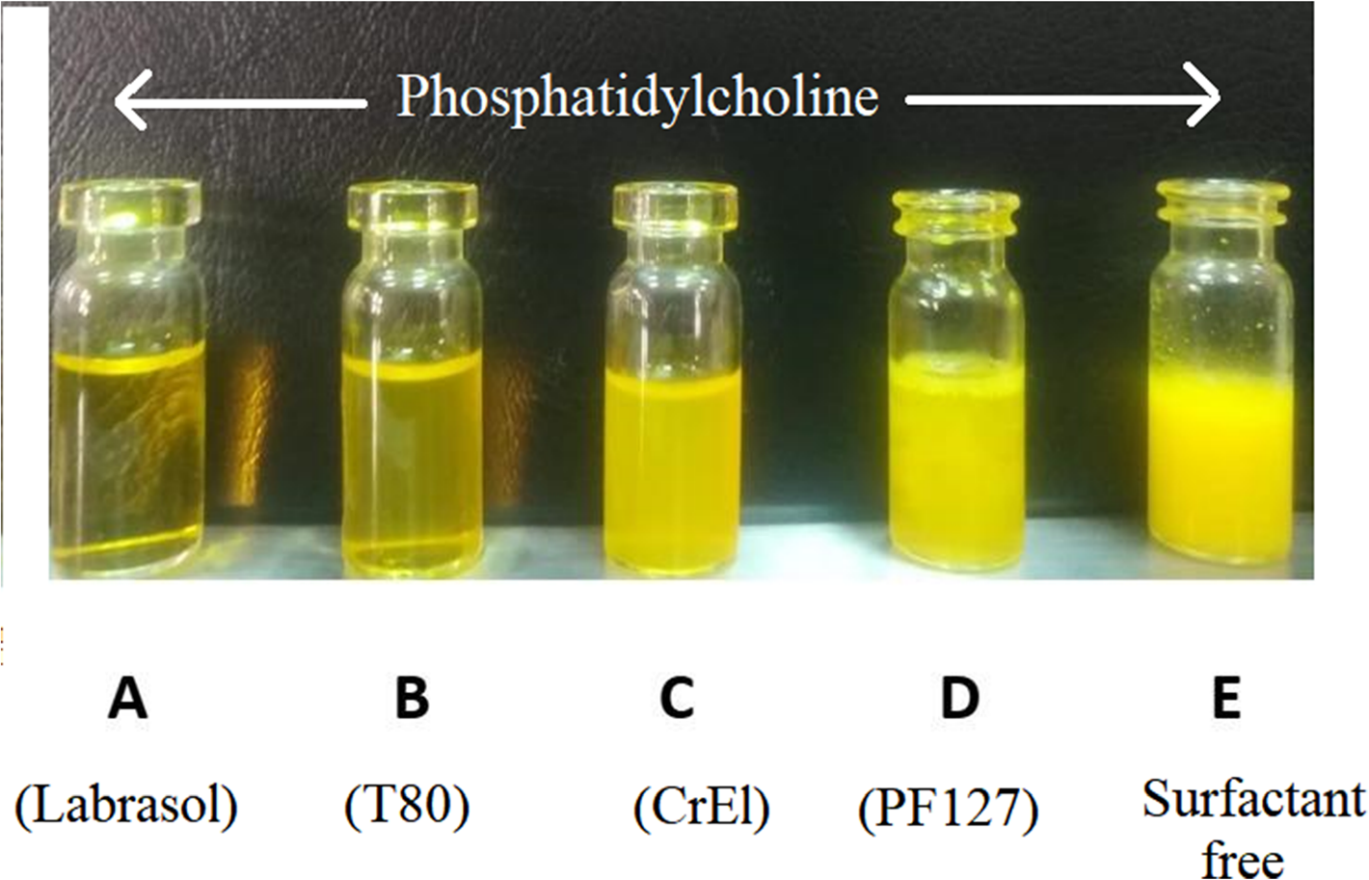
Photographs depicts the visual appearance of the prepared formulations: **A** F1, Labrasol based exhibiting transparent solution **B** F2, T80 based, with less transparent appearance **C** F3, CrEL based **D** F4, PF127 based **E** F5, surfactant free liposomal dispersion

Four nonionic surfactants (Labrasol^®^, T80, CrEL and PF127) were studied for the potential formation of bicelles with 1-α-phosphatidylcoline. Labrasol^®^ is a polyethylene glycol (PEG) derivative of medium chain triglyceride of capric and caprylic acid (a saturated polyglycolyzed C8-C10 glyceride) that is also known as caprylocaproyl macrogolglycerides. It is widely used in self-nanoemulsifying drug delivery systems as it possesses great solubilizing capacity and undergoes spontaneous self-emulsification. Furthermore, Labrasol^®^ enhance membrane fluidity and improve the permeability of many drugs (39). T80 is a hydrophilic surfactant composed of polyoxyethylene sorbitan with an oleic acid tail. It has been reported to have the ability of bicelles formation as it depicts critical micelle concentration which is much lower than that of the short-chain phospholipids or ionic surfactants (5,40). CrEL is widely used as a surfactant in nanoemulsion formulations, it is a polyoxyethylene derivative of castor oil with a branched alkyl chain structure (41). PF127 belongs to pluronics or poloxamers class; they are synthetic tri-block copolymers of poly(ethylene oxide)-poly(propylene oxide)-poly(ethylene oxide) (PEO-PPO-PEO). They have amphiphilic properties due to the presence of both hydrophobic and hydrophilic moieties. Their capability of interacting with hydrophobic surfaces and biological membranes promoted their use as surfactants (42). Among the investigated surfactants, only T80 was previously reported in literature to form bicelles. Herein, Fig. 2 displays the visual appearance of the prepared formulations. It is observable that in case of F1, labrasol^®^ formed isotropic clear solution of curcumin, while F2 that was formulated with T80 exhibited almost translucent appearance. This observations indicate that the solubility of curcumin in water was greatly enhanced in both F1 and F2, as depicted in Fig. 2A and B, respectively. Additionally, CrEl formed slightly opaque homogenous dispersion (Fig. 2C) and PF127 formed a liposome like dispersion (Fig. 2D). On the other hand, phosphatidylcholine alone was able to form homogenous milky liposomal dispersion when mixed with water (Fig. 2E).

### Docking Studies

**Fig. 3:**
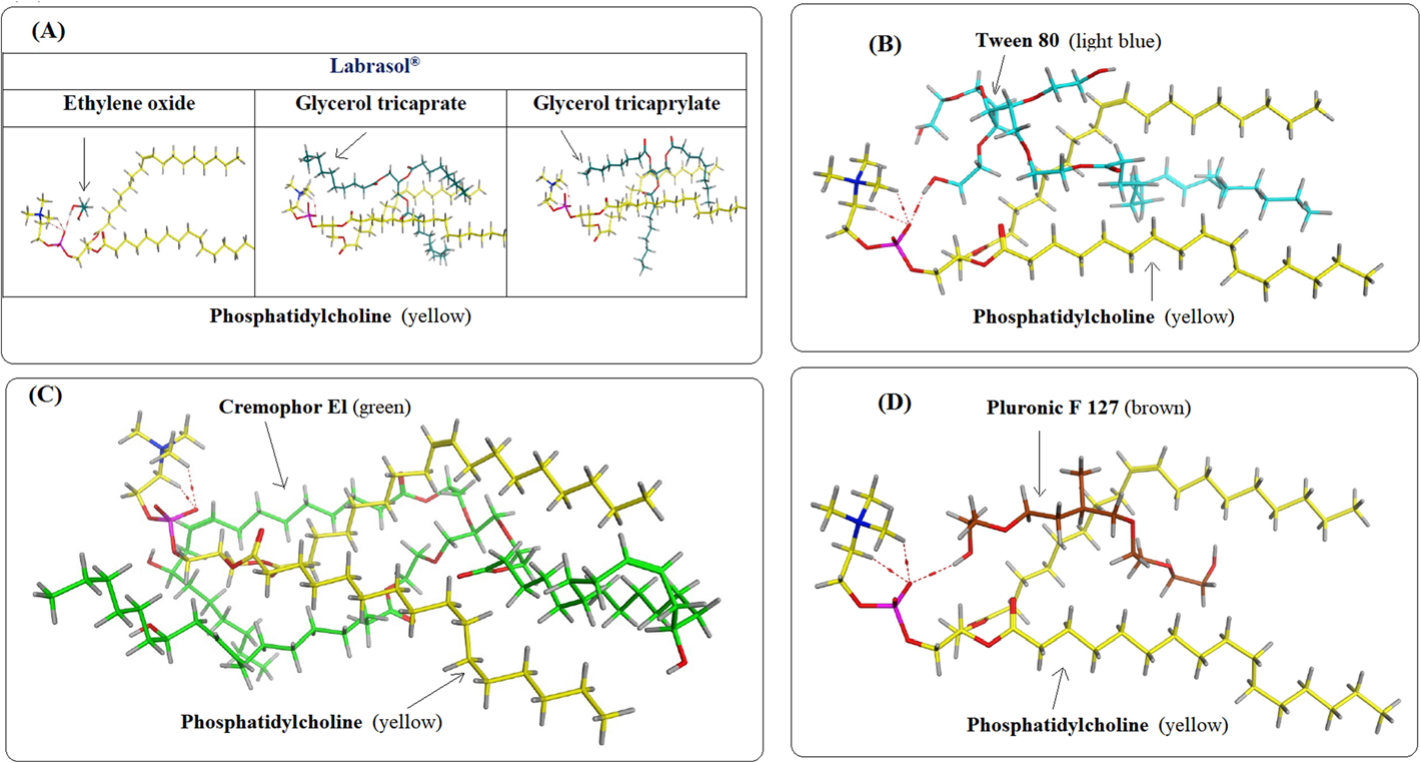
*In silico* study illustrates 3D binding interactions of phosphatidylcholine (yellow) to: **A** ethylene oxide, glycerol tricaprate, and glycerol tricaprylate (turquoise), respectively **B** T80 (light blue) **C** CrEL (green) **D** 3D binding interactions of PF127 (brown); Red dash represents H-bonds

Molecular docking was performed to provide the structural insight and to identify the potential intermolecular interaction of each surfactant with phosphatidylcholine during nanostructures formation; this can present a deep understanding of bicelle formation and the stability of the formulation. Analyzing the molecular docking results for the examined four nonionic surfactants (Labrasol^®^, T80, CrEL, and PF127) to phosphatidylcholine revealed a number of observations as follows:

- Labrasol^®^ components (ethylene oxide, glycerol tricaprate, and glycerol tricaprylate) were docked separately to phosphatidylcholine as discussed before. It is noticeable in Fig. 3A, that ethylene oxide formed H-bonds with the phosphate group of phosphatidylcholine, while glycerol tricaprate and glycerol tricaprylate oriented around its hydrophobic tails indicating the promising stability through Van der Waals bonds. This can be an interesting prediction of bicelles formation with labrasol^®^.In that case we envision that capric/caprylic glycerides of labrasol^®^ were aligned to contribute in the rim formation, as they do not interact with the hydrophilic head of phosphatidylcholine and the only observable interaction is with the hydrophobic tails of phosphatidylcholine. Moreover, PEG molecules are directed toward the hydrophilic heads of phosphatidylcholine.
- On the other hand, T80 also formed H-bonds with the phosphate group of phosphatidylcholine and its remaining structure oriented in between the hydrophobic tails of phosphatidylcholine indicating a large number of Van der Waals interactions as well (Fig. 3B). This gave us insight of the possible parallel alignment of the T80 molecules toward phosphatidylcoline molecules since the molecular interactions occur between both the hydrophobic and hydrophilic portions of both molecules.
- CrEL exhibited a great folding around the whole molecule of phosphatidylcholine referring to a large number of hydrophobic interactions (Fig. 3C). This great affinity of CrEL to phosphatidylcholine may exclude the possibility of bicelles formation as from the attained results it is expected that the surfactant molecules are aligned together with phosphatidylcholine molecules in an alternative manner and this will hinder the selective arrangement of surfactant molecules in the rim region.
- Furthermore, PF127 exhibited H-bond formation with the phosphate group of phosphatidylcholine and its remaining moiety oriented in between its hydrophobic tails indicating the previously mentioned Van der Waals interactions (Fig. 3D), hence it may favor mixed micelles formation.

Additionally, the values for binding energy of each surfactant with phosphatidylcholine were obtained and used as an indicative tool to assess the stability of the formed formulations as the greater the binding energy, the more stable the intermolecular interaction between molecules (43). The values of binding energy were -3.12, -5.82, -5.39, -6.24, -7.90, -4.34 in case of ethylene oxide, glycerol tricaprate, glycerol tricaprylate, T80, CrEL and PF127, respectively indicating the stability of the formed formulation. It is noticeable that CrEL has the highest binding affinity with phosphatidylcholine excluding the possibility of bicelles formation. Based on the aforementioned results concerning the interactions of the four tested nonionic surfactants to phosphatidylcholine, it is obvious that there is a great affinity between them as well as a crucial role of the phosphate group of phosphatidylcholine in these interactions, except in case of CrEL. This may help in understanding the mechanism of bicelles formation and could also help in the future design of more acceptable formulations with more desirable properties.

### Characterization of the Prepared Nanostructures

#### Morphological Examination of the Prepared Nanostructures

**Fig. 4:**
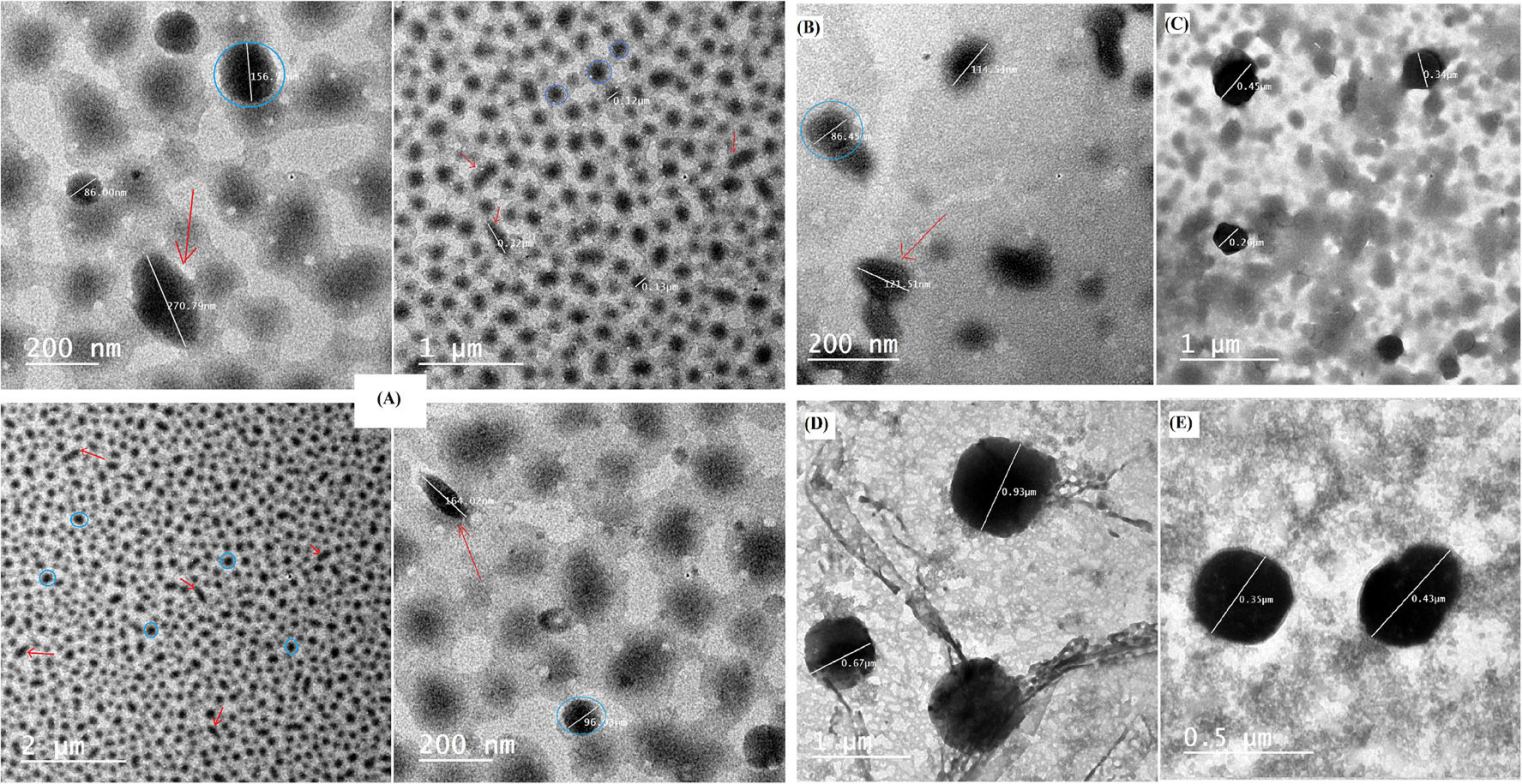
TEM micrographs at different magnifications of **A**: F1, labrasol based bicelles showing the side view of the discs (red arrows) and the round top view of the discs (Blue circles) depending on the orientation toward the incoming electron beam, **B**: F2, T80 based bicelles, **C**:F3, cremophor El based formulation, **D**: F4, PF127 based formulation, **E**: F5, surfactant free formulation

TEM micrographs of the investigated formulations are depicted in Fig. 4. The micrographs of F1 containing labrasol^®^ (Fig. 4A) confirmed the successful formation of bicelles, as predicted by the *in silico* study, as the sample exhibits spherical and oblong shapes, representing the top and the side views of bicelles, respectively. Labrasol^®^ is a mixture of 50% of mono- and di- fatty acid esters of PEG and 30% mono-, di- and triglycerides of saturated C6–C14 fatty acids (mainly C8 and C10 fatty acids) in addition to 20% of free PEG 400 (44). In water, polyoxyethylene containing nonionic surfactants exhibit a significant hydrophilic properties as it form coil-shaped meander structure. This structure is obtained when the oxygen atoms in ethylene oxide, which is the hydrophilic head group of polyoxyethylene nonionic surfactants, wrap the H_2_O molecules into the hydrophobic groups (-CH_2_) in order to achieve an efficient contact with H_2_O molecules (45). It is suggested that the hydration of the hydrophilic polyoxyethylene groups in labrasol^®^ causes formation of steric hindrance and a spontaneous curvature developing the rim of bicelles which shields the hydrophobic tails of phosphatidylcholine from water (4). It can be observed that labrasol^®^ bicelles are more distinct in shape than T80 bicelles (Fig. 4B), this is in agreement of the visual appearance of the formulations which exhibited the ability of labrasol^®^ to form more isotropic bicelles than that formed with T80. This is also in agreement with molecular docking results which suggested the ability of labrasol^®^ to form bicelles depicted from the absence of interaction of either glycerol tricaprate or glycerol tricaprylate with the hydrophilic head of phosphatidylcholine. On the other hand, there was a remarkable interaction between T80 and the hydrophilic head of phosphatidylcholine which suggests that there are some molecules alternatively aligned with phosphatidylcholine while the other molecules contributed to the formation of the rim of the bicelles. This suggestion is in compliance with the translucent appearance of F2. T80 is a sugar based surfactant that also belongs to the class of polyoxyethylene containing nonionic surfactants, it has large and big head groups so it form wide and dispersed hydrogen bond network (46,47). Hence, it is suggested that the addition of either labrasol^®^ or T80 to phospholipid allows denser packing of lipophilic chains due to the presence of steric hindrance between head groups, which permits formation and stabilization of the bicelles. On the other hand, the micrographs of F3 (CrEL) and F4 (PF127) which are shown in Fig. 4C and D, respectively, reveal the formation of almost spherical strucures indicating that bicelles could not be formed using these combinations. It may be suggested that both surfactants contributed with phosphatidylcholine in the formation of spherical nanostrucures. As in case of F3, the head groups of CrEL is composed of PEGs and glycerol ethoxylates, while the lipophilic chain portion consist of oxyethylated triglycerides of a long chain (C_18_) unsaturated ricinoleic acid. Though the lipophilic tails of both T80 and CrEL share the similar carbon chain length (C_18_) (48), the disability of CrEL to form bicelles like T80 may be justified by the presence of 3 chains of fatty acid attaching to PEG in CrEL, dissimilar to T80 which has only one fatty acid chain (oleic acid); thus the bulkier hydrophobic portion in CrEL may hinder the denser packing of the lipophilic tails that is required for rim formation. Additionally, the high binding affinity of CrEL to phosphatidylcholine and the H-bonding between the phosphate group of the latter hinder the formation of bicelles. In such case, it is suggested that the surfactants molecules favor being embedded along with phosphatidylcholine molecules. In case of F4, as mentioned before, PF127 is a synthetic tri-block copolymers of poly(ethylene oxide)-poly (propylene oxide)-poly(ethylene oxide), where the central poly(propylene oxide) and the two external poly (ethylene oxide) parts are the hydrophobic and hydrophilic portions of the surfactant, respectively. Here, PF127 can be hypothesized to form mixed micelles with phosphatidylcholine through incorporation of its hydrophilic ethylene oxide groups and the hydrophobic propylene oxide part into the hydrophilic and hydrophobic regions of the formed micelles. This can be attributed to the presence of two hydrophilic portions in PF127 molecule which facilitate its interaction with the hydrophilic heads of two molecules of phosphatidylcholine, favoring the alignment of the surfactant in a certain manner between phosphatidylcholine molecules. The result is in accordance with the previously reported formation of mixed micelles of PF127 with phospholipid in literature (49). Moreover, this incorporation can be the justification for the increase in particle size of the formed vesicles when compared to the liposome of F5 (surfactant free formulation) as shown in Fig. 4E, spherical vesicles (liposomes) were formed without surfactants addition.

#### PS, PDI, and ZP

Results of PS, PDI and ZP are listed in Table 1. The results of PS analysis revealed that labrasol^®^, T80 and CrEL formed nanostructures with phosphatidylcholine with PS 187.5 ± 31, 416.4 ± 30.8, 729.5 ± 85.7 nm, respectively. On the other hand, PF127 based formulation (F4) and the surfactant free formulation (F5) exhibited higher PS of 1167.3 ± 53.4 and 736.3 ± 39.5, respectively. It is observable that F1 and F2 exhibited smaller values of polydispersity index (0.462 ± 0.003 and 0.458 ± 0.026, respectively). On the other hand, F3, F4 and F5 depicted broader size distribution (0.639 ± 0 .066, 0.914 ± 0.041 and 0.818 ± 0.025, respectively). Results of ZP measurements revealed that the highest ZP value (-22.0 ± 0.2) attained in case of the surfactant free formulation (F5); this is attributable to the negative charges of the phosphate groups of phosphatidylcholine molecules. The values of ZP decreased upon mixing phosphatidylcholine with the surfactants in the following descending order: CrEl (F3), T80 (F2), PF127 (F4), labrasol^®^ (F1) as the values were -18.8 ± 0.2, -18.1 ± 1.8, -15.4 ± 0.4 and -6.8 ± 0.5, respectively. The decrease of ZP upon incorporation of nonionic surfactant suggested the existence of attractive forces due to the electrostatic interactions and hydrogen bond formation (50) between the oxygen atoms of phosphate groups of phosphatidylcholine and hydrogen atoms of the surfactants molecules. It is noticeable that the addition of labrasol^®^ exhibited significant decrease in ZP (from -22 to -6.8). This can be justified by the presence of free PEG molecules in the composition of labrasol^®^ (20%) (44), which form hydrogen bonds with the oxygen atoms of the phosphate groups as confirmed by the results of the molecular docking while the esterified PEGs content (total of 70%) contributes to the formation of bicelles rim. Although T80 molecules contain 2 hydroxyl groups available for hydrogen bond formation with the phosphate group of phosphatidylcholine, there is no marked drop in ZP values on the formulation (F2). This can be justified by the formation of bicelles where T80 molecules are contributing to the rim formation so the surfactant molecules are not adjacent to the hydrophilic head groups of phosphatidylcholine except at the rim region of the disk and the interacting portion with phosphatidylcholine as explained in the results of the molecular docking. In case of PF127 formulation (F4), the hydrogen bonds are formed between the ethylene oxide blocks and the phosphate groups of phosphatidylcholine in the formed mixed micelles thus decreasing the negative ZP. On the other hand, CrEl did not cause drop in ZP values, this is suggested to be due to the absence of free hydrogen atoms in the ethoxylated glycerol heads of CrEL which hinders hydrogen bond formation with the phosphate group of phosphatidylcholine molecule and thus the repulsive forces are retained.

#### Assessment of Transmittance Percentage

Assessment of transmittance percentage was done in order to judge the transparency of the prepared formulations and the ability of the investigated surfactant to produce isotropic bicelles upon interaction with phosphatidycholine. Results are listed in Table 1; it is noticeable that % transmittance was extremely variable between the formulations, as it ranged from 91.6 ± 1.1% to 1.8 ± 0.5%. The highest % transmittance was obtained with labrasol^®^-based formulation suggesting the formation of isotropic bicelles; the transparency decreased in the following order: labrasol^®^ > T80 > CrEL > PF127 with the lowest % transmittance was obtained in case of F5 (plain liposome formulation).

#### *In Vitro* Dissolution Study

**Fig. 5:**
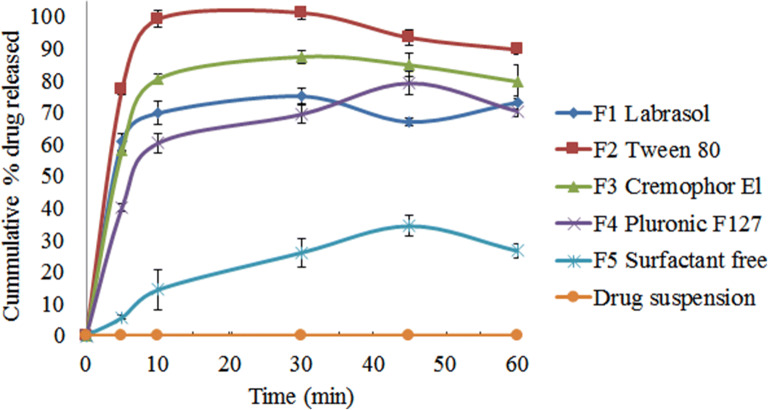
*In vitro* drug release profiles of the prepared formulations compared to curcumin suspension in water

In order to assess the effectiveness of the developed formulations to enhance the solubility of a typical extremely hydrophobic compound such as curcumin, non-sink conditions were selected to conduct the study. In such a non-sink condition, the formulations are expected to yield a temporarily supersaturated drug solutions in which the drug concentrations are significantly greater than the saturated solubility of the drug in the medium (51). Therefore, performing the dissolution studies in non-sink conditions has recently considered an important approach to judge the true performance of the formulations through assessing the supersaturation state and the liability for the drug precipitation (52). The dissolution profiles of curcumin from the developed formulations in addition to the drug suspension are illustrated in Fig. 5. Curcumin amounts of that dissolved from the drug suspension were negligible and not detected at all the investigated time intervals. Generally, there is a marked enhancement of the dissolution profiles of curcumin from all surfactants-containing formulations (F1, F2, F3, and F4) when compared to either surfactant-free formulation (F5) or the drug suspension; the % drug dissolved after 30 min in case of F1, F2, F3, and F4 were 75 ± 2.6, 101.2 ± 1.9, 87.4 ± 2.1, 69.4 ± 2.7 % , respectively, which are significantly higher (P < 0.05) than the % dissolved from F5 (26 ± 4.5%). It is noticeable that the bicelles formulation F2 (T80) showed the best dissolution profile with the maximum % drug dissolved of 99.2 ± 2.6 % in the first 10 min, the formulation retained the supersaturation till 30 min and a slight drug precipitation was observed at 45 min to yield % dissolved of 89.6±1.6 % after 1 h. On the other hand, Labrasol^®^ based bicelles (F1) exhibited high yet incomplete dissolution of curcumin as the maximum % of drug dissolved was 73.1±2 % though the drug was completely soluble in the form of an isotropic solution as depicted in Fig. 2A, this may be suggesting the occurrence of a slight variation in the bicelles structure upon dilution, in accordance with a previous report that stated the occurrence of transition in the disc structure upon certain dilution conditions to larger structures such as vesicles (53). This dilution effect is not observed in T80 bicelles and that is in agreement with the literature as it has been reported that T80 bicelles exhibited resistance to dilution (5). Generally, the observed improvement of the dissolution profile of curcumin from bicelles formulations (F1 and F2) can be attributed to the presence of the drug in the hydrophobic portion of the formed discs along with their small size. Further, the enhancement of dissolution from F3 and F4 may be attributed to the formed mixed micelles of small sizes. Overall, the surfactant contents of the formulations aided in ameliorating curcumin solubility.

#### *Ex Vivo* Permeability Test (Everted Gut Sac Model)

**Fig. 6:**
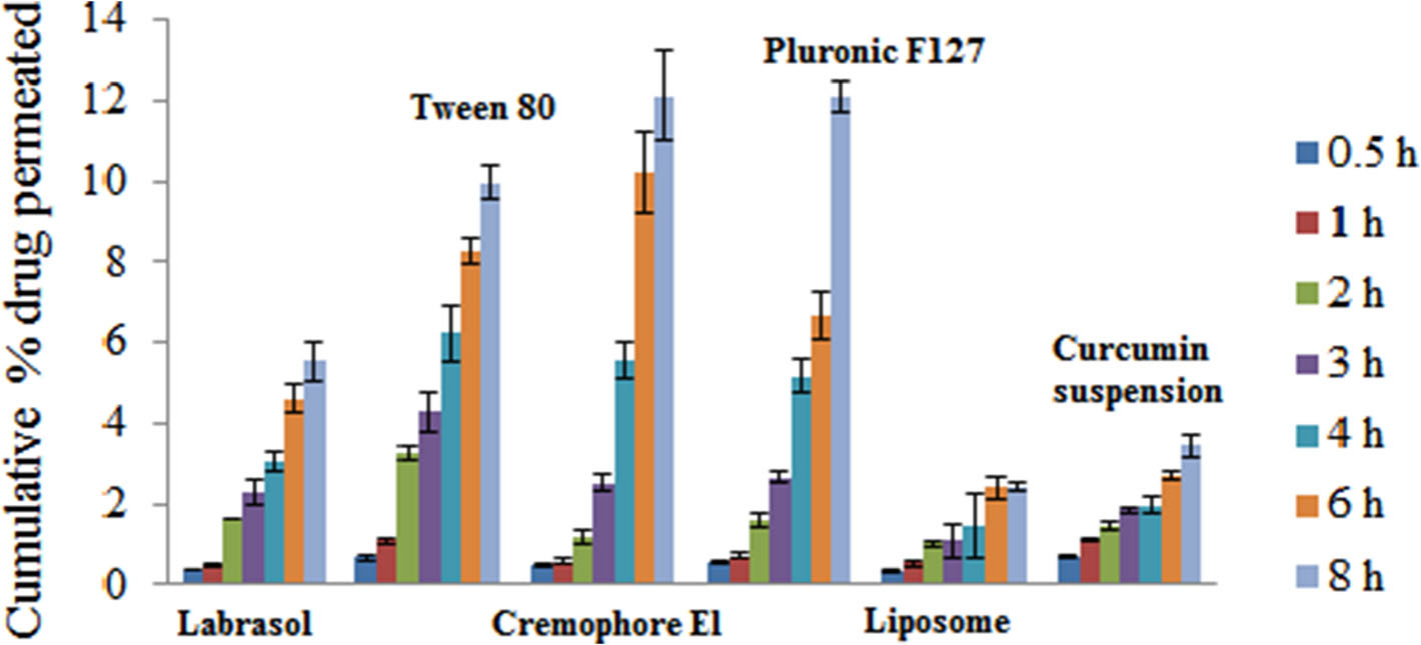
Results of *ex vivo* permeation study

Permeability assessment of drug through the gut is considered as a fundamental step to predict the drug absorption from the investigated formulations. Therefore, the everted gut sac model was utilized to compare the permeation of the investigated drug from the prepared nanostructures relative to both the drug suspension and the surfactant-free formulation (F5). It is noticeable from Fig. 6, that the cumulative drug permeated percentages in case of F1, F2, F3 and F4 were relatively higher than both the drug suspension and F5 starting from (3h) and reaching the maximum at the end of the study (8h). Different factors can affect the drug permeation rate like PS, hydrophilic-lipophilic balance (HLB) of the used surfactant, morphology and surface charge. The negatively charged mucus contains acid components including sulfonic acid and sialic acid justifying the net negative charge of the mucus. Hence, the uncharged and the negatively charged nanoparticles are able to penetrate across the mucus unlike the positively charged nanoparticles that cannot penetrate through mucus layer as a result of the ionic interactions (54). In compliance with this, our results revealed that F3 that exhibited the highest negative charge showed the highest drug absorption profile. Additionally, this can also be justified by the high HLB value of CrEL (14) as the direct relationship between the HLB value and the penetration enhancing effect of the used surfactant was previously reported (55, 56). Though the ZP value of F2 was higher than F4, the cumulative amount of drug absorbed from F4 was higher than that of F2, this can be attributed to the higher HLB value of PF127 than that of T80 (22, 15; respectively). In case of F1, the enhancement of drug permeation was compromised between two antagonizing factors including the thin shape of the formed discs along with their smaller size which enhance their ability to penetrate and pass the narrow gaps (57), at the same time the incomplete drug absorption can be attributed to the lower negative ZP value of the formula (-6.8±0.5). It is noteworthy to point to the role of the 20 % free PEG content of labrasol in interacting with and shielding of the phosphate group of the bicelles hydrophilic heads and hence decreasing the ZP value of F1. This can also justify the superiority of F2 over F1 in improving the drug permeation. Generally, the improvement of the *ex vivo* drug absorption from F1, F2, F3 and F4 comparing with F5 is justified by their high surfactant content.

#### *In Vitro* Cytotoxicity and Antiviral Activity Against SARS-CoV-2

**Fig. 7:**
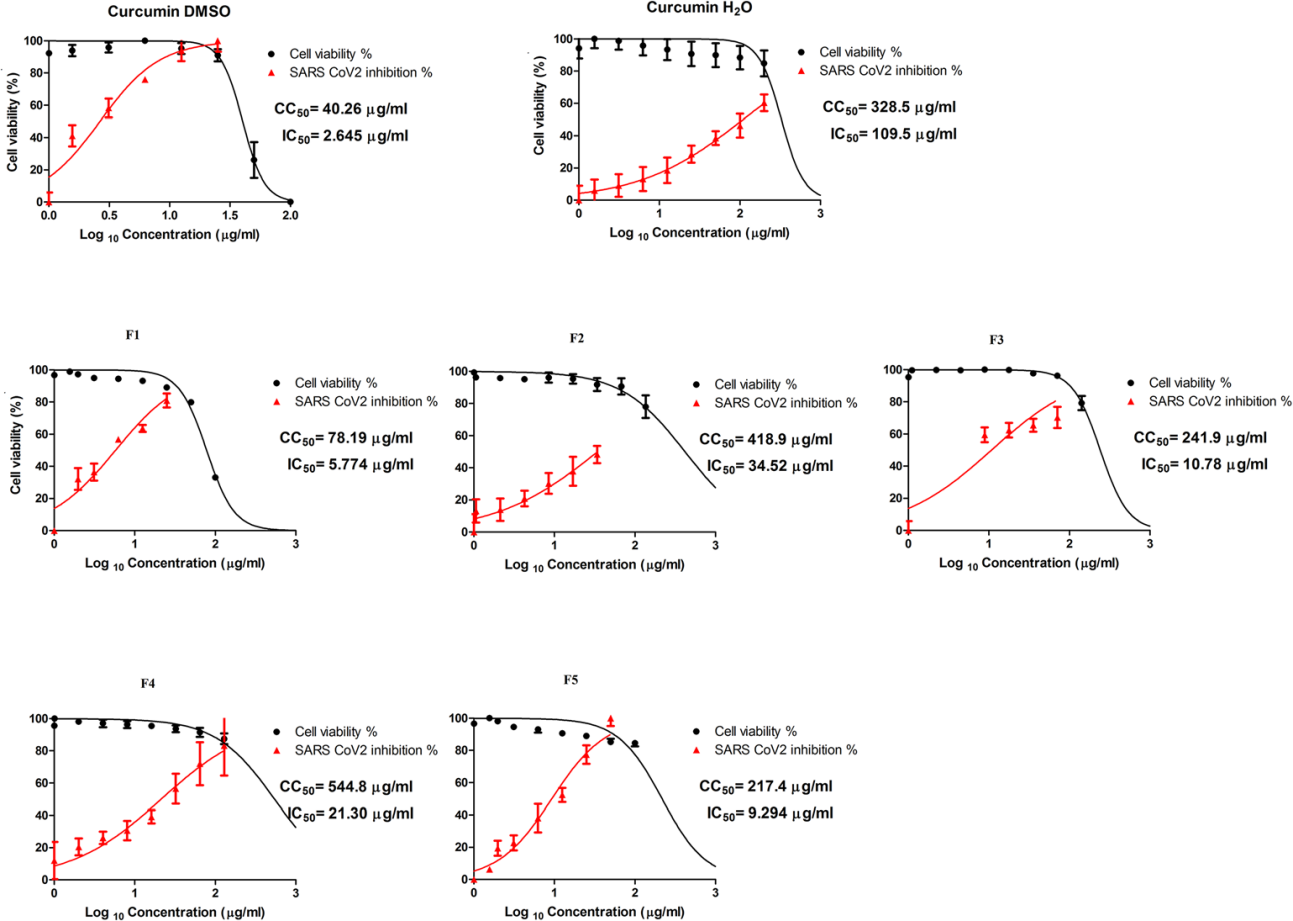
Results of in vitro study of cytotoxicity and antiviral activity of the prepared formulations against SARS-CoV-2

The cytotoxicity of curcumin solution in DMSO, curcumin suspension in water, F1, F2, F3, F4, and F5 in Vero-E6 cells were determined by MTT assay and the result revealed that the CC_50_ values were 40.26, 328.5, 78.19, 418.9, 241.9, 544.8, 217.4 μg/ml, respectively (as shown in Fig. 7). The antiviral activities of curcumin solution in DMSO, curcumin suspension in water, F1, F2, F3, F4 and F5 were investigated relying on the dose–response and the results depicted that the IC_50_ values were 2.645, 109.5, 5.774, 34.52, 10.78, 21.30, 9.294 μg/ml, respectively. Assessment of cytotoxicity was performed to ensure that the cell death was due to the inhibitory effect of the investigated curcumin samples on SARS-CoV-2 virus and that the decrease in viral titre was not related to cell death and to determine the safe concentrations for IC_50_ estimation. Taking into account both CC_50_ and IC_50_ values, the selectivity index (SI) was determined as the ratio of CC_50_ to IC_50_ of each sample; the SI values of curcumin solution in DMSO, curcumin suspension in water, F1, F2, F3, F4 and F5 were 15.22, 3, 13.54, 12.13, 22.44, 25.58, and 23.39 respectively. Generally, it is observable that the lowest SI value was attained with curcumin suspension as the extremely low solubility of the drug in aqueous media hinders its antiviral activity. On the other hand, there was a statistically significant increase of SI of curcumin in all the fabricated formulations when compared to the drug suspension (p<0.05) signifying an augmented therapeutic activity of the formulations. Both bicelles formulations (F1 and F2) ameliorated the SI of curcumin more than the drug suspension by 4.5 and 4 fold; this can be attributed to the enhanced dissolution of curcumin from the formulated bicelles with higher concentrations of the drug available in the media to exert its antiviral activity. Moreover, the structure of the bicelles is suggested to enhance the rapid internalization of the drug inside the cells. This is more noticeable in case of the isotropic bicelles solution (F1) as it demonstrated the highest antiviral potency and the highest cytotoxicity indicated by the lowest IC_50_ and CC_50_ values, respectively, among the other formulations (F2, F3, F4 and F5). Further, the fomrulations that exhibited slower release profiles of curcumin (F4 and F5), demonstrated higher SI values than both bicelles formulations (F1 and F2). This observation may be explained by the fact that SI is a compromised value which considers both the toxicity of the material to normal cells as well as to the virus. Hence, the slowly release manner of F4 and F5 provides the effective antiviral drug concentrations gradually in a way that does not harm the normal cells. As an exception to this justification, CrEL containing formulation (F3) that exhibited high dissolution profile of curcumin yet exhibited high SI value this may indicate that the dissolution rate of the drug from the formula was optimally compromised with the cytotoxicity of the formulation. Although, liposomes and mixed micelles formulations exhibited better selectivity indices than bicelles formulations, it is important not to neglect the significant overall improvement of bicelles of curcumin delivery and antiviral activity when compared to drug suspension. Overall, the aforementioned advantages of bicelles formulations in improving the dissolution and subsequently the absorption of curcumin along with augmenting its therapeutic activity would make them a promising drug carrier; particularly when a solution dosage form is required. Finally, our developed nanostructures are amenable to overcome the drawbacks that hinder utilization of curcumin as an effective antiviral in the management of COVID 19.

## CONCLUSION

Non-ionic surfactants were studied for substitution of short chain phospholipids to form bicelles. Four nonionic surfactants including labrasol^®^, T80, CrEL and PF127 were studied. the molecular docking of each surfactant with phosphatidylcholine presented a useful tool to predict the bicelles formation which is further confirmed by formulation and characterization. Among the tested surfactants, only labrasol and T80 were able to form bicelles. However, labrasol bicelles are more isotropic than the bicelles formed with T80. . The improvement of the dissolution profile of the extremely hydrophobic curcumin from the formed bicelles presents the bicelles as a promising oral drug delivery system for hydrophobic drugs that further can be administered in an isotropic form. Generally, all the designed formulae were able to enhance the oral delivery of curcumin. However, labrasol containing bicelles were inferior to other nanostructures in the enhancement of curcumin permeability. This was justified by the lower ZP value coming from its content of free PEG. Hence, further studies are recommended to investigate other surfactants for their viability to form bicelles where the negatively charged phosphate group of phosphatidylcholine is kept unshielded. Finally, the therapeutic activity of curcumin as an antiviral therapy against SARS CoV-2 was improved using all the developed nanostructures. Consequently, this study presents various possible formulations that can be utilized for future preclinical and clinical studies against COVID-19.
